# Hospital characteristics associated with low-value care in public hospitals in New South Wales, Australia

**DOI:** 10.1186/s12913-020-05625-4

**Published:** 2020-08-14

**Authors:** Tim Badgery-Parker, Sallie-Anne Pearson, Adam G. Elshaug

**Affiliations:** 1 Faculty of Medicine and Health, School of Public Health, Menzies Centre for Health Policy, Charles Perkins Centre, The University of Sydney, Sydney, Australia; 2 Research Fellow, Centre for Health Systems and Safety Research, Australian Institute of Health Innovation, Macquarie University, Sydney, Australia; 3 Centre for Big Data Research in Health, University of New South Wales, Kensington, Australia; 4 The Brookings Institution, USC-Brookings Schaeffer Initiative for Health Policy, Washington, D.C., USA

## Abstract

**Background:**

Rates of low-value care vary between hospitals in New South Wales, Australia. Understanding factors associated with this variation will help in understanding the drivers of low-value care and in planning initiatives to reduce low-value care.

**Methods:**

For eight low-value procedures, we used Poisson regression of the number of low-value episodes at each hospital to assess the association between low-value care and hospital characteristics. We also used hierarchical clustering on the low-value procedures used and their rates at each hospital to try to identify groups of hospitals with higher or lower rates of low-value care across multiple procedures.

**Results:**

Some hospital characteristics, such as hospital peer group and proportion of total episodes that involve the specific procedure, showed associations for some procedures, but none were consistent across all eight procedures. We clustered hospitals into five groups, but low-value care rates did not differ much between these groups.

**Conclusion:**

Available hospital variables show little association with rates of low-value care and no patterns across different low-value procedures. We need to investigate factors within hospitals, such as clinician knowledge and beliefs about low-value care.

## Background

Choosing Wisely [1] and similar movements have highlighted various tests and procedures that are low value and not expected to provide a net benefit in specific patient groups. Efforts have been made to measure how much these tests and procedures are used in practice, with measurement programs in various countries, including the United States [2–4], Canada [5, 6], Austria [7], and Australia [8–11].

The prevalence of low-value care varies by region [3, 6] and by hospital [10, 12]. Factors that may contribute to this variation include aspects of the health system and the population [3, 6]. For example, the ratio of specialist to primary care practitioners is a significant factor in the United States [3] and Alberta, Canada [6]. For population factors, race and poverty rate are significant in the United States [3], while age, sex, and neighourhood income are significant in Alberta [6].

In New South Wales, Australia’s most populous state, there is considerable interhospital variation in the prevalence of low-value care [10]. In a previous analysis, we used multilevel models to assess the contribution of Local Health District (LHD) and hospital of treatment and patient’s area of residence to the variation in low-value care prevalence [13]. LHDs are administrative regions that make funding and policy decisions for all hospitals in the region. Hence, differences in policy could explain differences in low-value care prevalence. However, we found the LHD contributed little to the total variation [13]. We also found the patient’s area of residence explained little of the variation; most variation is at the hospital level [13]. Therefore, for this analysis we narrow the focus to further examine variation between hospitals.

A full understanding of interhospital variation in low-value care is likely to require research within hospitals to examine local policies, access to technological infrastructure, attitudes and knowledge of individual clinicians, and aspects of hospital “culture” [13], but such data are not yet available. This exploratory analysis was conducted to assess how much currently available administrative data can tell us about interhospital variation in low-value care.

## Methods

### Data and setting

Inpatient care in Australia is provided in public and private hospitals. In New South Wales, the most populous state, there are 221 public facilities and 210 private facilities [14], although many of these hospitals do not provide the services we are studying. We were not given access to private hospital data for this analysis.

Our data included all admissions to public hospitals in New South Wales for the period 1 July 2010 to 30 June 2017. The data include patient demographic information (e.g., age, sex, area of residence), clinical information (e.g., procedures performed and diagnoses assigned), and administrative information (e.g., private or public patient, emergency or elective care) for all inpatient episodes at public hospitals in NSW. Outpatient encounters are not included in this dataset.

### Low-value care indicators

We have previously developed indicators based on admitted patient data to identify low-value care involving 27 procedures [9, 10]. Many of these procedures have very low numbers of low-value episodes in our setting [10]. For this study, we selected eight procedures with annual low-value numbers ranging from about 80 (renal artery angioplasty) to 1762 (endoscopy for dyspepsia in people < 55 years old) (Table 1). The lower limit of about 80 was arbitrarily chosen to provide sufficient low-value episodes per hospital and per parameter to obtain reasonable model estimates.

**Table 1:** Definitions of low-value procedures

Procedure (recommendation source)	Denominator	Numerator
Carotid endarterectomy for asymptomatic high-risk patients with limited life expectancy (CWA, CWC, CWUS)	Episodes involving people aged 18 years or older with diagnosis of occlusion and stenosis of carotid artery or procedure of carotid endarterectomy with no stroke or focal neurological symptoms recorded in the episode, and ASA score 4–5 or (age ≥ 75 and ASA 3) and not emergency care or admitted through emergency department	Denominator episodes involving carotid endarterectomy
Colonoscopy for constipation in people aged < 50 years (CWC)	Episodes involving people aged 18–49 years with constipation and no diagnoses of anaemia, weight loss, family or personal history of cancer of digestive system, or personal history of other diseases of digestive system recorded in previous 12 months	Denominator episodes involving colonoscopy
Endoscopy for dyspepsia in people aged < 55 years (CWC)	Episodes involving people aged 18–54 years with dyspepsia and no diagnoses of dysphagia, iron deficiency anaemia, other nutritional anaemia, abnormal weight loss, family or personal history of cancer of digestive system, or personal history of peptic ulcer in the previous 12 months	Denominator episodes involving endoscopy
Endovascular repair of abdominal aortic aneurysm (CWC)	Episodes involving people aged 18 years or older with diagnosis of abdominal aortic aneurysm and ASA score 4–5 or (age ≥ 75 with ASA score 3), and not emergency care or admitted through emergency department	Denominator episodes involving endovascular repair of aneurysm
Abdominal hysterectomy for benign disease (vs laparoscopic or vaginal approach) (Committee on Gynecologic Practice)	Episodes involving women aged 18 years or older having hysterectomy with no caesarean, cancer, endometriosis, or pelvic peritoneal adhesions recorded in the episode	Denominator episodes involving abdominal approach
Arthroscopic lavage and debridement of knee for osteoarthritis or degenerate meniscal tears (CWUS, NICE)	Episodes involving people aged 55 or older with diagnosis of gonarthrosis or meniscal derangements and no diagnosis of ligament strain or damage or diagnosis of septic (pyogenic) arthritis recorded in the episode	Denominator episodes involving knee arthroscopy
Renal artery angioplasty or stenting (HealthPACT)	Episodes involving people aged 18 years or older with diagnosis of renovascular hypertension, atherosclerosis of renal artery, hypertensive kidney disease, or hypertensive heart and kidney disease and no diagnosis of fibromuscular dystrophy or pulmonary oedema	Denominator episodes involving renal artery angioplasty or stenting
Percutaneous coronary intervention with balloon angioplasty or stent placement for stable coronary disease (CWUS)	Episodes involving people aged 18 years or older with diagnosis of coronary disease except angina in any episode 6–18 months before index episode and no episodes involving coronary disease in the 6 months before index episode and not emergency care or admitted through emergency department	Denominator episodes involving percutaneous coronary intervention

*CWA* Choosing Wisely Australia, *CWC* Choosing Wisely Canada, *CWUS* Choosing Wisely United States, *NICE* National Institute of Health and Care Excellence, *HealthPACT* Health Policy Advisory Committee on Technology

### Analysis

Our analysis was in two parts. First, for each procedure, we modelled the number of low-value episodes at each hospital per year (so the unit of observation is the hospital-year). Using RStan [15] through the brms package [16], we estimated multilevel Poisson models with a random intercept term for hospitals to account for correlation within hospitals between years. Explanatory variables were year, the hospital peer group (a classification of similar hospitals based on total volume of episodes and types of service provided; see Additional file 1) [17], the proportion of female patients at the hospital in each year, the proportion of episodes involving patients aged over 65 years at the hospital in each year, whether the hospital was in a rural or metropolitan LHD, and the proportion of total episodes at the hospital that involved the procedure being modelled in each year. The age, sex, and rural variables were intended to capture differences in the hospital catchment populations. Rural status also reflects access to specialist care [18]. The proportion of total episodes that involved the relevant procedure was included because hospitals that do a procedure more often may also be doing more low-value procedures [13]. We included the number of episodes involving the procedure at each hospital in each year as an offset term. We fit models with each explanatory variable individually for unadjusted results, and then included all variables in a single model to produce estimates adjusted for all variables.

Secondly, we used hierarchical clustering to assess if there are groups of hospitals with high or low rates of low-value care across multiple procedures. Our approach was to build a typology of hospitals based on the low-value procedures they performed and the rates of those procedures, and then assess if the hospital groups differ on other characteristics. We created a series of indicators of whether each procedure was provided (0 if not provided) because not all procedures are provided at all hospitals. Because the proportion of low value services varied substantially between procedures, we standardised the observed proportions to the range 0–1, by subtracting the minimum and dividing by the range of observed proportions for each procedure. We used the indicators of whether the procedure was provided and the standardised proportions of each low-value procedure in the hierarchical clustering. That is, we grouped hospitals on the procedures they provided and the low-value rates of those procedures. We then compared these groups on hospital characteristics, including total volume, number of the procedures provided, percentage of female patients, percentage of patients aged > 65 years, and rural or metropolitan location.

## Results

Additional file 2 contains a summary of the hospital characteristics for each of the low-value procedures included in this study.

### Hospital characteristics associated with low-value care

**Fig. 1:**
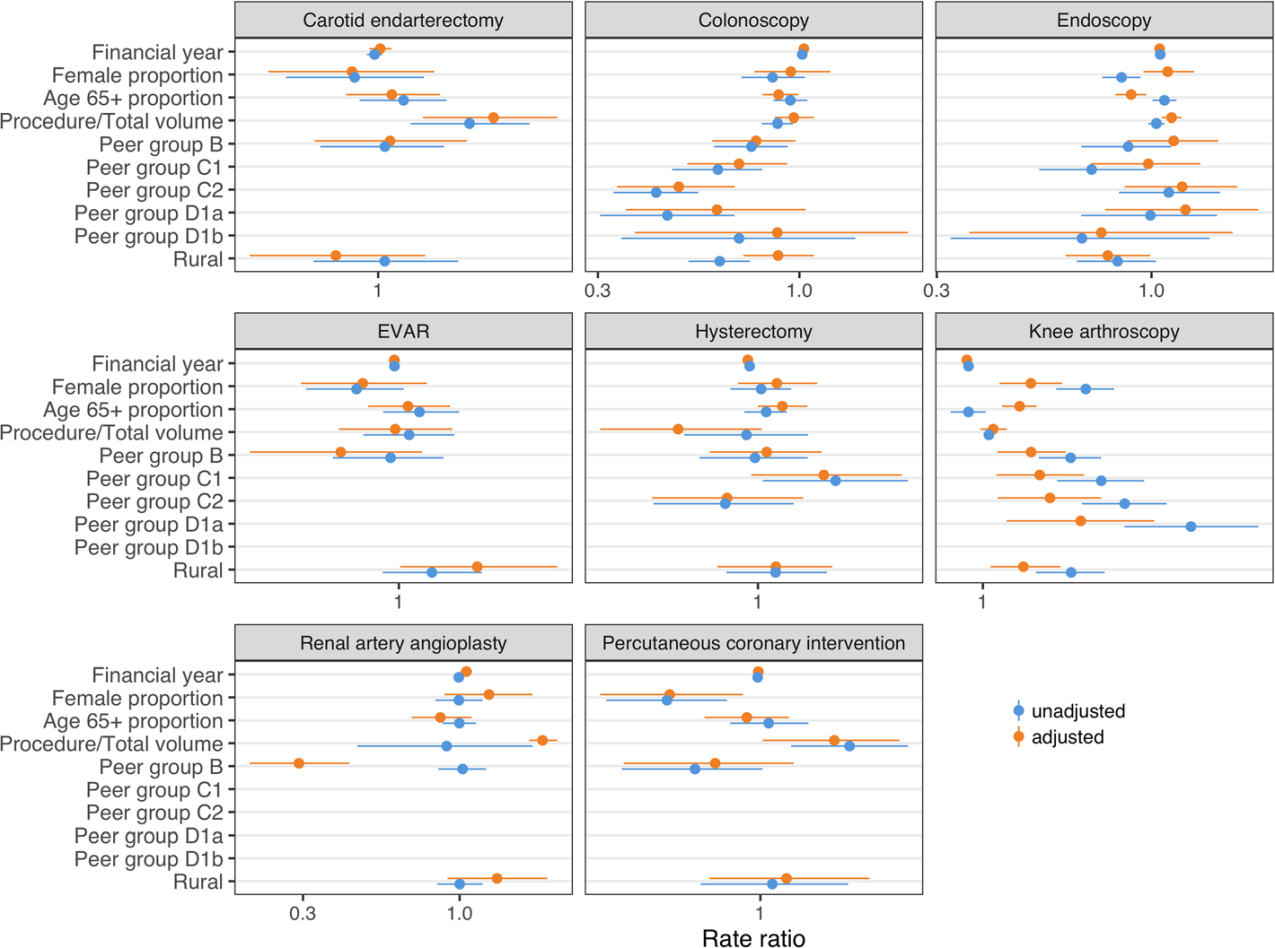
Rate ratios for low-value care by various hospital characteristics. Unadjusted results are from univariable mixed effects Poisson models. Adjusted results are from multivariable mixed effects Poisson models, and so are adjusted for all other variables included in the model

In general, the hospital characteristics available for this analysis showed little clear association with rates of low-value care, with no clear pattern across different low-value procedures (Fig. 1). Hospitals where the relevant procedure accounted for a higher proportion of all procedures at the hospital were more likely to do low-value carotid endarterectomy, knee arthroscopy, renal artery angioplasty, and percutaneous coronary intervention (PCI). In contrast, hospitals with more hysterectomies as a proportion of all procedures were less likely to perform abdominal (low-value) hysterectomies. Compared with principal referral hospitals, peer group B hospitals (major hospitals) were less likely to do low-value renal artery angioplasty and PCI. Low-value knee arthroscopy appeared more likely at smaller community hospitals than principal referral hospitals, but adjustment for other variables reduced or reversed this effect.

Low-value colonoscopy was less likely at smaller hospitals, but a similar pattern was not observed for endoscopy, although in our dataset about 40% of patients under 50 years who receive colonoscopy also receive endoscopy.

Rural hospitals had lower rates of low-value colonoscopy, endoscopy, and carotid endarterectomy, but higher rates of low-value knee arthroscopy, endovascular repair of abdominal aortic aneurysm (EVAR), and renal artery angioplasty.

### Clustering hospitals by low-value care

**Fig. 2:**
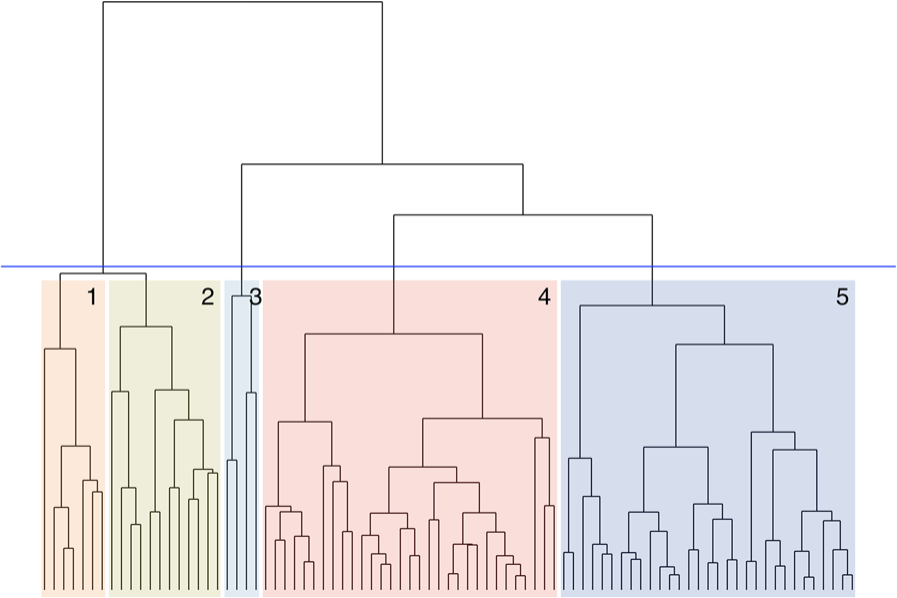
Hierarchical clustering of hospitals by low-value care services and rates

After viewing the hierarchical clustering tree, we chose to group the hospitals into five clusters (Fig. 2). Table 2 summarises the characteristics of the hospitals in the five groups derived from hierarchical clustering. We did not observe a significant difference in the rates of low-value care between clusters; hence there is no clear association between hospital characteristics and low-value care across multiple procedures. However, the large hospitals in cluster 1 and the small hospitals in cluster 5 had the lower rates of low-value care than the other clusters.

**Table 2:** Characteristics of hospital clusters

Cluster	Number of hospitals, and by peer group	Number of hospitals in rural area	Median number of the 8 procedures provided (IQR)	Median total hospital volume (IQR)	Median proportion of separations involving women (IQR)	Median proportion of separation involving people > 65 (IQR)	Median observed proportion of low-value care (IQR)	Median standardised proportion of low-value care (IQR)	Notes
1	7 A: 7	1	8 (8–8)	80,425 (60779–81,297)	0.51 (0.50–0.51)	0.39 (0.37–0.42)	0.14 (0.12–0.15)	0.28 (0.28–0.33)	These major hospitals all do all 8 procedures.
2	12 A: 6 B: 6	4	7 (6–7)	38,307 (29144–55,569)	0.49 (0.48–0.51)	0.43 (0.40–0.49)	0.17 (0.15–0.20)	0.41 (0.35–0.47)	None of these hospitals do renal artery angioplasty. Four hospitals do not do EVAR, but all hospitals do all the other procedures.
3	4 A: 2 C2: 1 D1a: 1	2	1 (1–1.25)	2561 (1998–6191)	0.55 (0.51–0.65)	0.42 (0.31–0.46)	0.22 (0.18–0.32)	0.31 (0.26–0.48)	Two hospitals do hysterectomy and three do knee arthroscopy. Only one hospital does both.
4	31 B: 15 C1: 12 C2: 4	17	4 (4–5)	15,759 (9267–20,835)	0.51 (0.49–0.54)	0.43 (0.35–0.46)	0.15 (0.11–0.18)	0.38 (0.33–0.41)	These hospitals do not do carotid endarterectomy, EVAR, or renal artery angioplasty. Ten hospitals do PCI. All hospitals do all the remaining procedures.
5	31 A: 1 B: 1 C1: 2 C2: 19 D1a: 7 D1b: 1	29	3 (2–3)	3155 (2140–5273)	0.53 (0.50–0.54)	0.47 (0.40–0.54)	0.10 (0.05–0.12)	0.28 (0.23–0.39)	These are generally small rural hospitals, although two large rural hospitals are also included. They all do colonoscopy and endoscopy. Thirteen do knee arthroscopy, and six do hysterectomy.

Clusters 1 and 2 are predominantly large metropolitan hospitals; cluster 1 comprises only principal referral hospitals, while cluster 2 has both principal referral and major hospitals. Cluster 1 hospitals are larger and provide all the eight potentially low-value procedures, while cluster 2 hospitals each only provide six or seven of the procedures, and renal artery angioplasty is not provided by any of them. The low-value care rate was slightly higher in cluster 2 than in cluster 1.

Cluster 3 consists of two rural hospitals, a principal referral hospital, and a specialist women’s hospital. Each of these provides only one of the eight services. This group had the lowest rate of low-value care.

Clusters 4 and 5 are small hospitals. Cluster 4 includes smaller metropolitan hospitals, and rural community hospitals, while cluster 5 is primarily small rural hospitals. Cluster 5 hospitals do fewer of the procedures and have lower rates of low-value care compared with cluster 4.

## Discussion

Hospitals vary in their rates of low-value care in New South Wales [10], and internationally [19, 20], but there is little information about the factors that contribute to this variation. In this study, we examined several hospital characteristics but found no general associations across eight low-value procedures. Some characteristics, such as hospital peer group and proportion of total episodes that involve the specific procedure, showed associations for some procedures, but none were consistent across all eight procedures. We also clustered the hospitals by the procedures they perform and the rates of low-value care across the procedures to try to identify groups that have higher or lower rates of low-value care generally, with little success.

Our results imply low-value care is not a general property of a hospital in NSW, but varies by procedure within hospital. Low-value care needs to be reported and investigated by procedure type. Combining low-value estimates for multiple procedures at the hospital level will not convey a true picture of low-value care in NSW, especially as most hospitals do not provide all the procedures being measured. This is in contrast to the United States, where multiple low-value indicators have been combined to form the Johns Hopkins Overuse Index (JHOI) [20]. The main reason for this difference is population. The JHOI uses 20 tests and procedures with > 800 events across the United States [20], while we have only eight procedures with 80–1800 events in NSW.

In general, NSW hospitals that have relatively higher volumes of a particular procedure tended to have higher low-value rates for that procedure. Direct measures of low-value care, such as ours, can be used to monitor actual rates of low-value care, as well as associated costs or downstream consequences [21]. However, data limitations meant we could not develop measures for most recommendations [9]. The results presented here suggest higher overall rates of a procedure may be a useful indirect indicator of hospitals where low-value care occurs for recommendations that cannot be adapted for direct measurement. It is important that such indirect measures only be used to target further investigation, as efforts to simply reduce rates may lead to undertreatment of patients for whom a procedure is appropriate [22].

As the hospital characteristics we examined here are not strongly associated with low-value care, we need to look elsewhere for explanations of the variation in rates between hospitals. A Norwegian study found important variation in patient safety culture at the ward level [23], and our results support this possibility that low-value care also varies between small units within hospitals. The most obvious factor to consider is individual treating doctors, as it is they who decide (in consultation with the patient) what treatment to provide. Group (by specialty or ward) level practice norms may also develop independently within a hospital, resulting in low-value care ‘hotspots’ that reflect a department within a hospital as opposed to the hospital itself. This is a critical insight for quality improvement initiatives. Whether a hospital has high or low rates of low-value care likely depends on the choices of the doctors who work there, and will depend on their knowledge of and agreement with the recommendations and underlying evidence [24].

## Conclusion

NSW hospitals do not appear to have high or low rates of low-value care across multiple procedures. Instead, low-value rates of each procedure need to be examined separately. Basic characteristics of the hospitals and patient populations do not strongly associate with low-value care. Understanding the drivers of low-value care is likely to require mixed method studies within hospitals. In particular, examining the knowledge and attitudes of the clinicians who provide these procedures may be more likely to provide explanations of the hospital variation in rates of low-value care.

## Supplementary information

**Additional file 1.** Definitions of hospital peer groups used in New South Wales.

**Additional file 2.** Summary of the hospital characteristics for each low-value procedure.

**Additional file 3.** Multivariable models and output log.

## Data Availability

The data that support the findings of this study are held by the NSW Ministry of Health and restrictions apply to the use of these data under the *Health Records and Information Privacy Act 2002* (NSW). On reasonable request, the authors will assist in preparing a data request and ethics application to the NSW Population and Health Services Research Ethics Committee.

## Abbreviations

EVAR: Endovascular repair of abdominal aortic aneurysm
JHOI: Johns Hopkins Overuse Index
LHD: Local Health District
NSW: New South Wales
PCI: Percutaneous coronary intervention
