# Supplementary material for: Hospital characteristics associated with low-value care in public hospitals in New South Wales, Australia
Source: BMC Health Serv Res. 2020 Aug 14;20:750. doi: 10.1186/s12913-020-05625-4 (PMC7427854; doi:10.1186/s12913-020-05625-4)
Supplement: Supplementary file 1 — Additional file 1. Definitions of hospital peer groups used in New South Wales. [file 12913_2020_5625_MOESM1_ESM.docx]

Definitions of hospital peer groups

Adapted from NSW Ministry of Health. NSW Hospital Peer Groups 2016 [Internet]. Sydney: NSW Health; 2016. Available from: https://www1.health.nsw.gov.au/pds/ActivePDSDocuments/IB2016_013.pdf

A1: Principal referral

2011: Acute hospitals, treating more than 30,000 acute casemix weighted separations per year, with average cost weight > 1.3.

2014: Greater than 35,000 acute weighted separations and offering highly specialised services

B: Major hospitals

2011: Establishments treating 10,000 to 30,000 acute casemix weighted separations, including a rural and non-rural referral role.

2014: B1: 17,000 to 35,000 acute weighted separations and availability of one or more specialist services requiring specific infrastructure or average NWAU (National Weighted Activity Unit) per separation ≥ 1.25.

2014: B2: 10,000 to 35,000 acute weighted separations and no specialist services requiring specific infrastructure or average NWAU per separation < 1.25.

C1: District group 1

2011: Acute hospitals treating 5000 to 10,000 acute casemix weighted separations per year.

2014: 4000 to 10,000 acute weighted separations per year.

C2: District group 2

2011: Acute hospitals treating 2000 to 5000 acute casemix weighted separations per year.

2014: 2000 to 4000 acute weighted separations per year.

D1a: Community acute with surgery

Acute hospitals treating 200 to 2000 acute casemix weighted separations per year with more than 2% of their acute weighted separations being surgical.

D1b: Community acute without surgery

2011: Acute hospitals treating 200 to 2000 acute casemix weighted separations per year with less than 2% of acute weighted separations being surgical and less than 40% of total bed-days being acute outlier, subacute or non-acute bed-days.

2014: ≤ 2000 acute separations and < 2% surgery, or < 200 total separations

Note: other peer groups exist but are not relevant for the procedures in this study.

Note: peer groups were revised in 2014 (halfway through our data period), but all hospitals in this study remained grouped with the same peers. However, the 2014 revision also separated peer group B into two groups. For consistency of grouping in this analysis, we chose to keep peer groups B1 and B2 as a single peer group B.
