# Supplementary material for: Hospital characteristics associated with low-value care in public hospitals in New South Wales, Australia
Source: BMC Health Serv Res. 2020 Aug 14;20:750. doi: 10.1186/s12913-020-05625-4 (PMC7427854; doi:10.1186/s12913-020-05625-4)
Supplement: Supplementary file 3 — Additional file 3. Multivariable models and output log. [file 12913_2020_5625_MOESM3_ESM.pdf]

Model hospital rates of lvc, with linear trends

```
library(here)
```

```
## here() starts at /Users/tbad5666/repositories/phd-analyses-clean
```

```
library(timbpfns)
library(tidyverse)
```

```
## ── Attaching packages ─────────────────────────────────────────
tidyverse 1.2.1 ──
```

```
## ✔ ggplot2 3.0.0     ✔ purrr   0.2.5
## ✔ tibble  1.4.2     ✔ dplyr   0.7.6
## ✔ tidyr   0.8.1     ✔ stringr 1.3.1
## ✔ readr   1.1.1     ✔ forcats 0.3.0
```

```
## ── Conflicts ──────────────────────────────────────────── tidyv
erse_conflicts() ──
## ✖ dplyr::filter() masks stats::filter()
## ✖ dplyr::lag()    masks stats::lag()
```

```
library(brms)
```

```
## Loading required package: Rcpp
```

```
## Loading 'brms' package (version 2.4.0). Useful instructions
## can be found by typing help('brms'). A more detailed introduction
## to the package is available through vignette('brms_overview').
## Run theme_set(theme_default()) to use the default bayesplot theme.
```

```
library(plotly)
```

```
##
## Attaching package: 'plotly'
```

```
## The following object is masked from 'package:ggplot2':
##
##     last_plot
```

```
## The following object is masked from 'package:stats':
##
##     filter
```

```
## The following object is masked from 'package:graphics':
##
##     layout
```

```
options(mc.cores = parallel::detectCores())
datadir <- "C:/users/tbadg/documents/downloaded_data/"
set.seed(20170919)

processed_data = readRDS(here("hosp-variation", "data", "processed-data.rds"))
alldta <- bind_rows(processed_data, .id = "lvservice") %>%
  mutate(finyr=factor(cYear, levels=-6:0, labels=c("1011", "1112", "1213", "1314",
                                                 "1415", "1516", "1617")))

prior_b <- set_prior("student_t(4, 0, 5)", class = "b")
```

PCI

```
m_pci <- brm(nLVC_N ~ cYear + peer_group + I((srvproptot - mean(srvproptot)) / 10)
+ I((femaleprop - .5) * 10) + I((age65 - .5) * 10) + rural +
  (1  | facility_identifier) + offset(log(N)),
family = "poisson", data = alldta[alldta$lvservice=="pci",], prior = prior_b,
iter = 750, chains = 4, control = list(max_treedepth = 13, adapt_delta = 0.9)
)
```

```
## Compiling the C++ model
```

```
## Start sampling
```

```
summary(m_pci)
```

```
##  Family: poisson
##   Links: mu = log
## Formula: nLVC_N ~ cYear + peer_group + I((srvproptot - mean(srvproptot))/10) + I
((femaleprop - 0.5) * 10) + I((age65 - 0.5) * 10) + rural + (1 | facility_identifie
r) + offset(log(N))
##    Data: alldta[alldta$lvservice == "pci", ] (Number of observations: 211)
## Samples: 4 chains, each with iter = 750; warmup = 375; thin = 1;
##          total post-warmup samples = 1500
##
## Group-Level Effects:
## ~facility_identifier (Number of levels: 34)
##               Estimate Est.Error l-95% CI u-95% CI Eff.Sample Rhat
## sd(Intercept)     0.54      0.14     0.32     0.85        504 1.01
##
## Population-Level Effects:
##                               Estimate Est.Error l-95% CI u-95% CI
## Intercept                        -4.52      0.23    -4.96    -4.06
## cYear                            -0.01      0.02    -0.05     0.02
## peer_groupB                      -0.29      0.34    -0.97     0.29
## IsrvproptotMmeansrvproptotD10     0.45      0.27    -0.10     1.00
## IfemalepropM0.5MU10              -0.58      0.29    -1.15    -0.02
## Iage65M0.5MU10                   -0.07      0.18    -0.42     0.28
## ruralR                            0.18      0.33    -0.45     0.85
##                               Eff.Sample Rhat
## Intercept                            704 1.00
## cYear                               1500 1.00
## peer_groupB                          613 1.00
## IsrvproptotMmeansrvproptotD10       1048 1.00
## IfemalepropM0.5MU10                  703 1.00
## Iage65M0.5MU10                       709 1.00
## ruralR                               677 1.00
##
## Samples were drawn using sampling(NUTS). For each parameter, Eff.Sample
## is a crude measure of effective sample size, and Rhat is the potential
## scale reduction factor on split chains (at convergence, Rhat = 1).
```

Knee arthroscopy

```
m_knart <- brm(nLVC_N ~ cYear + peer_group + I((srvproptot - mean(srvproptot)) / 1
0) + I((femaleprop - .5) * 10) + I((age65 - .5) * 10) + rural +
  (1  | facility_identifier) + offset(log(N)),
family = "poisson", data = alldta[alldta$lvservice=="knee_arthroscopy",], prior = p
rior_b,
iter = 500, chains = 4, control = list(max_treedepth = 17)
)
```

```
## Compiling the C++ model
```

```
## Start sampling
```

```
summary(m_knart)
```

```
##  Family: poisson
##   Links: mu = log
## Formula: nLVC_N ~ cYear + peer_group + I((srvproptot - mean(srvproptot))/10) + I
((femaleprop - 0.5) * 10) + I((age65 - 0.5) * 10) + rural + (1 | facility_identifie
r) + offset(log(N))
##    Data: alldta[alldta$lvservice == "knee_arthroscopy", ] (Number of observation
s: 443)
## Samples: 4 chains, each with iter = 500; warmup = 250; thin = 1;
##          total post-warmup samples = 1000
##
## Group-Level Effects:
## ~facility_identifier (Number of levels: 67)
##               Estimate Est.Error l-95% CI u-95% CI Eff.Sample Rhat
## sd(Intercept)     0.67      0.07     0.54     0.83        405 1.00
##
## Population-Level Effects:
##                                  Estimate Est.Error l-95% CI u-95% CI
## Intercept                           -2.40      0.16    -2.70    -2.09
## cYear                               -0.08      0.01    -0.09    -0.07
## peer_groupB                         -0.03      0.13    -0.29     0.20
## peer_groupC1                        -0.03      0.25    -0.49     0.45
## peer_groupC2                        -0.12      0.25    -0.62     0.38
## peer_groupD1a                       -0.17      0.37    -0.89     0.56
## IsrvproptotMmeansrvproptotD10        0.17      0.05     0.07     0.27
## IfemalepropM0.5MU10                  0.36      0.09     0.17     0.52
## Iage65M0.5MU10                       0.03      0.06    -0.09     0.15
## ruralR                               0.53      0.20     0.15     0.91
##                                  Eff.Sample Rhat
## Intercept                               556 1.00
## cYear                                  1000 1.00
## peer_groupB                             785 1.00
## peer_groupC1                            359 1.01
## peer_groupC2                            349 1.01
## peer_groupD1a                           501 1.01
## IsrvproptotMmeansrvproptotD10           896 1.00
## IfemalepropM0.5MU10                    1000 1.00
## Iage65M0.5MU10                          686 1.00
## ruralR                                  428 1.01
##
## Samples were drawn using sampling(NUTS). For each parameter, Eff.Sample
## is a crude measure of effective sample size, and Rhat is the potential
## scale reduction factor on split chains (at convergence, Rhat = 1).
```

Renal artery angioplasty

```
m_renal <- brm(nLVC_N ~ cYear + peer_group + I((srvproptot - mean(srvproptot)) * 1
0) + I((femaleprop - .5) * 10) + I((age65 - .5) * 10) + rural +
  (1  | facility_identifier),
family = "poisson", data = alldta[alldta$lvservice=="renal_angioplasty",], prior =
prior_b,
iter = 750, chains = 4, control = list(max_treedepth = 13, adapt_delta = 0.95)
)
```

```
## Compiling the C++ model
```

```
## Start sampling
```

```
summary(m_renal)
```

```
##  Family: poisson
##   Links: mu = log
## Formula: nLVC_N ~ cYear + peer_group + I((srvproptot - mean(srvproptot)) * 10) +
I((femaleprop - 0.5) * 10) + I((age65 - 0.5) * 10) + rural + (1 | facility_identifi
er)
##    Data: alldta[alldta$lvservice == "renal_angioplasty", ] (Number of observatio
ns: 130)
## Samples: 4 chains, each with iter = 750; warmup = 375; thin = 1;
##          total post-warmup samples = 1500
##
## Group-Level Effects:
## ~facility_identifier (Number of levels: 25)
##               Estimate Est.Error l-95% CI u-95% CI Eff.Sample Rhat
## sd(Intercept)     0.27      0.08     0.14     0.46        492 1.01
##
## Population-Level Effects:
##                                  Estimate Est.Error l-95% CI u-95% CI
## Intercept                            1.66      0.17     1.31     2.01
## cYear                                0.05      0.02     0.01     0.09
## peer_groupB                         -1.25      0.24    -1.70    -0.78
## IsrvproptotMmeansrvproptotMU10       0.61      0.05     0.50     0.71
## IfemalepropM0.5MU10                  0.20      0.21    -0.23     0.62
## Iage65M0.5MU10                      -0.14      0.14    -0.42     0.15
## ruralR                               0.33      0.23    -0.10     0.78
##                                  Eff.Sample Rhat
## Intercept                              1085 1.00
## cYear                                  1500 1.00
## peer_groupB                            1092 1.00
## IsrvproptotMmeansrvproptotMU10         1465 1.00
## IfemalepropM0.5MU10                    1314 1.00
## Iage65M0.5MU10                         1110 1.00
## ruralR                                 1088 1.00
##
## Samples were drawn using sampling(NUTS). For each parameter, Eff.Sample
## is a crude measure of effective sample size, and Rhat is the potential
## scale reduction factor on split chains (at convergence, Rhat = 1).
```

EVAR

```
m_evar <- brm(nLVC_N ~ cYear + peer_group + I(srvproptot - mean(srvproptot)) + I((f
emaleprop - .5) * 10) + I((age65 - .5) * 10) + rural +
  (1  | facility_identifier) + offset(log(N)),
family = "poisson", data = alldta[alldta$lvservice=="evar",], prior = prior_b,
iter = 500, chains = 4, control = list(max_treedepth = 13, adapt_delta = 0.95)
)
```

```
## Compiling the C++ model
```

```
## Start sampling
```

```
summary(m_evar)
```

```
##  Family: poisson
##   Links: mu = log
## Formula: nLVC_N ~ cYear + peer_group + I(srvproptot - mean(srvproptot)) + I((fem
aleprop - 0.5) * 10) + I((age65 - 0.5) * 10) + rural + (1 | facility_identifier) +
offset(log(N))
##    Data: alldta[alldta$lvservice == "evar", ] (Number of observations: 119)
## Samples: 4 chains, each with iter = 500; warmup = 250; thin = 1;
##          total post-warmup samples = 1000
##
## Group-Level Effects:
## ~facility_identifier (Number of levels: 21)
##               Estimate Est.Error l-95% CI u-95% CI Eff.Sample Rhat
## sd(Intercept)     0.06      0.05     0.00     0.18        238 1.00
##
## Population-Level Effects:
##                              Estimate Est.Error l-95% CI u-95% CI Eff.Sample
## Intercept                       -0.80      0.09    -0.98    -0.63       1000
## cYear                           -0.00      0.01    -0.03     0.02       1000
## peer_groupB                      0.05      0.14    -0.24     0.32        529
## IsrvproptotMmeansrvproptot       0.03      0.11    -0.17     0.24        865
## IfemalepropM0.5MU10             -0.08      0.12    -0.32     0.18       1000
## Iage65M0.5MU10                   0.07      0.07    -0.06     0.21        721
## ruralR                           0.08      0.12    -0.14     0.30        470
##                              Rhat
## Intercept                    1.00
## cYear                        1.00
## peer_groupB                  1.00
## IsrvproptotMmeansrvproptot 1.00
## IfemalepropM0.5MU10          1.00
## Iage65M0.5MU10               1.00
## ruralR                       1.01
##
## Samples were drawn using sampling(NUTS). For each parameter, Eff.Sample
## is a crude measure of effective sample size, and Rhat is the potential
## scale reduction factor on split chains (at convergence, Rhat = 1).
```

Hysterectomy

```
m_hyst <- brm(nLVC_N ~ cYear + peer_group + I((srvproptot - mean(srvproptot)) / 10)
+ I((femaleprop - .5) * 10) + I((age65 - .5) * 10) + rural +
  (1  | facility_identifier) + offset(log(N)),
family = "poisson", data = alldta[alldta$lvservice=="hysterectomy",], prior = prior
_b,
iter = 500, chains = 4, control = list(max_treedepth = 13)
)
```

```
## Compiling the C++ model
```

```
## Start sampling
```

```
summary(m_hyst)
```

```
##  Family: poisson
##   Links: mu = log
## Formula: nLVC_N ~ cYear + peer_group + I((srvproptot - mean(srvproptot))/10) + I
((femaleprop - 0.5) * 10) + I((age65 - 0.5) * 10) + rural + (1 | facility_identifie
r) + offset(log(N))
##    Data: alldta[alldta$lvservice == "hysterectomy", ] (Number of observations: 4
42)
## Samples: 4 chains, each with iter = 500; warmup = 250; thin = 1;
##          total post-warmup samples = 1000
##
## Group-Level Effects:
## ~facility_identifier (Number of levels: 67)
##               Estimate Est.Error l-95% CI u-95% CI Eff.Sample Rhat
## sd(Intercept)     0.44      0.06     0.35     0.57         35 1.05
##
## Population-Level Effects:
##                                  Estimate Est.Error l-95% CI u-95% CI
## Intercept                           -1.47      0.13    -1.73    -1.21
## cYear                               -0.04      0.01    -0.05    -0.03
## peer_groupB                         -0.10      0.14    -0.39     0.16
## peer_groupC1                         0.16      0.21    -0.25     0.56
## peer_groupC2                        -0.26      0.21    -0.69     0.17
## IsrvproptotMmeansrvproptotD10       -0.29      0.20    -0.66     0.12
## IfemalepropM0.5MU10                  0.10      0.09    -0.09     0.28
## Iage65M0.5MU10                       0.10      0.06    -0.02     0.21
## ruralR                               0.14      0.15    -0.14     0.44
##                                  Eff.Sample Rhat
## Intercept                               160 1.00
## cYear                                  1000 1.00
## peer_groupB                             174 1.01
## peer_groupC1                            249 1.01
## peer_groupC2                            149 1.02
## IsrvproptotMmeansrvproptotD10           638 1.01
## IfemalepropM0.5MU10                     627 1.00
## Iage65M0.5MU10                          431 1.00
## ruralR                                  214 1.02
##
## Samples were drawn using sampling(NUTS). For each parameter, Eff.Sample
## is a crude measure of effective sample size, and Rhat is the potential
## scale reduction factor on split chains (at convergence, Rhat = 1).
```

Carotid endarterectomy

```
m_cea <- brm(nLVC_N ~ cYear + peer_group + I(srvproptot - mean(srvproptot)) + I((fe
maleprop - .5) * 10) + I((age65 - .5) * 10) + rural +
  (1  | facility_identifier) + offset(log(N)),
family = "poisson", data = alldta[alldta$lvservice=="carotid_endarterectomy",], pri
or = prior_b,
iter = 500, chains = 4, control = list(max_treedepth = 13, adapt_delta = 0.9)
)
```

```
## Compiling the C++ model
```

```
## Start sampling
```

```
summary(m_cea)
```

```
##  Family: poisson
##   Links: mu = log
## Formula: nLVC_N ~ cYear + peer_group + I(srvproptot - mean(srvproptot)) + I((fem
aleprop - 0.5) * 10) + I((age65 - 0.5) * 10) + rural + (1 | facility_identifier) +
offset(log(N))
##    Data: alldta[alldta$lvservice == "carotid_endarterectomy (Number of observati
ons: 153)
## Samples: 4 chains, each with iter = 500; warmup = 250; thin = 1;
##          total post-warmup samples = 1000
##
## Group-Level Effects:
## ~facility_identifier (Number of levels: 24)
##               Estimate Est.Error l-95% CI u-95% CI Eff.Sample Rhat
## sd(Intercept)     0.28      0.08     0.15     0.48        219 1.01
##
## Population-Level Effects:
##                            Estimate Est.Error l-95% CI u-95% CI Eff.Sample
## Intercept                     -1.59      0.16    -1.91    -1.27        563
## cYear                          0.01      0.02    -0.03     0.05        892
## peer_groupB                    0.05      0.20    -0.35     0.42        603
## IsrvproptotMmeansrvproptot     0.45      0.17     0.14     0.79        655
## IfemalepropM0.5MU10           -0.11      0.21    -0.51     0.31        673
## Iage65M0.5MU10                 0.08      0.13    -0.17     0.35        660
## ruralR                        -0.17      0.24    -0.59     0.31        335
##                            Rhat
## Intercept                  1.00
## cYear                      1.00
## peer_groupB                1.00
## IsrvproptotMmeansrvproptot 1.00
## IfemalepropM0.5MU10        1.00
## Iage65M0.5MU10             1.00
## ruralR                     1.02
##
## Samples were drawn using sampling(NUTS). For each parameter, Eff.Sample
## is a crude measure of effective sample size, and Rhat is the potential
## scale reduction factor on split chains (at convergence, Rhat = 1).
```

Colonoscopy

```
m_colon <- brm(nLVC_N ~ cYear + peer_group + I((srvproptot - mean(srvproptot)) / 1
0) + I((femaleprop - .5) * 10) + I((age65 - .5) * 10) + rural +
  (1  | facility_identifier) + offset(log(N)),
family = "poisson", data = alldta[alldta$lvservice=="colonoscopy",], prior = prior_
b,
iter = 500, chains = 4, control = list(max_treedepth = 13)
)
```

```
## Compiling the C++ model
```

```
## Start sampling
```

```
summary(m_colon)
```

```
##  Family: poisson
##   Links: mu = log
## Formula: nLVC_N ~ cYear + peer_group + I((srvproptot - mean(srvproptot))/10) + I
((femaleprop - 0.5) * 10) + I((age65 - 0.5) * 10) + rural + (1 | facility_identifie
r) + offset(log(N))
##    Data: alldta[alldta$lvservice == "colonoscopy", ] (Number of observations: 58
1)
## Samples: 4 chains, each with iter = 500; warmup = 250; thin = 1;
##          total post-warmup samples = 1000
##
## Group-Level Effects:
## ~facility_identifier (Number of levels: 87)
##               Estimate Est.Error l-95% CI u-95% CI Eff.Sample Rhat
## sd(Intercept)     0.38      0.05     0.28     0.49        311 1.00
##
## Population-Level Effects:
##                                Estimate Est.Error l-95% CI u-95% CI
## Intercept                         -3.04      0.13    -3.30    -2.78
## cYear                              0.02      0.01    -0.00     0.04
## peer_groupB                       -0.27      0.14    -0.52     0.01
## peer_groupC1                      -0.42      0.18    -0.74    -0.07
## peer_groupC2                      -0.78      0.19    -1.14    -0.40
## peer_groupD1a                     -0.66      0.32    -1.30    -0.02
## peer_groupD1b                     -0.27      0.48    -1.25     0.65
## IsrvproptotMmeansrvproptotD10     0.02      0.07    -0.12     0.14
## IfemalepropM0.5MU10               0.01      0.12    -0.24     0.24
## Iage65M0.5MU10                   -0.05      0.06    -0.17     0.08
## ruralR                           -0.15      0.12    -0.41     0.07
##                                Eff.Sample Rhat
## Intercept                             164 1.03
## cYear                                1000 1.00
## peer_groupB                           354 1.01
## peer_groupC1                          323 1.02
## peer_groupC2                          441 1.01
## peer_groupD1a                         413 1.01
## peer_groupD1b                         732 1.00
## IsrvproptotMmeansrvproptotD10         575 1.00
## IfemalepropM0.5MU10                   590 1.01
## Iage65M0.5MU10                        409 1.01
## ruralR                                374 1.00
##
## Samples were drawn using sampling(NUTS). For each parameter, Eff.Sample
## is a crude measure of effective sample size, and Rhat is the potential
## scale reduction factor on split chains (at convergence, Rhat = 1).
```

Endoscopy

```
m_endos <- brm(nLVC_N ~ cYear + peer_group + I((srvproptot - mean(srvproptot)) / 1
0) + I((femaleprop - .5) * 10) + I((age65 - .5) * 10) + rural +
  (1  | facility_identifier) + offset(log(N)),
family = "poisson", data = alldta[alldta$lvservice=="endoscopy",], prior = prior_b,
iter = 500, chains = 4, control = list(max_treedepth = 13)
)
```

```
## Compiling the C++ model
```

```
## Start sampling
```

```
summary(m_endos)
```

```
##  Family: poisson
##   Links: mu = log
## Formula: nLVC_N ~ cYear + peer_group + I((srvproptot - mean(srvproptot))/10) + I
((femaleprop - 0.5) * 10) + I((age65 - 0.5) * 10) + rural + (1 | facility_identifie
r) + offset(log(N))
##    Data: alldta[alldta$lvservice == "endoscopy", ] (Number of observations: 574)
## Samples: 4 chains, each with iter = 500; warmup = 250; thin = 1;
##          total post-warmup samples = 1000
##
## Group-Level Effects:
## ~facility_identifier (Number of levels: 86)
##               Estimate Est.Error l-95% CI u-95% CI Eff.Sample Rhat
## sd(Intercept)     0.51      0.06     0.42     0.63        146 1.03
##
## Population-Level Effects:
##                               Estimate Est.Error l-95% CI u-95% CI
## Intercept                        -2.08      0.13    -2.35    -1.81
## cYear                             0.05      0.00     0.04     0.06
## peer_groupB                       0.05      0.15    -0.26     0.33
## peer_groupC1                     -0.09      0.19    -0.44     0.29
## peer_groupC2                      0.26      0.18    -0.08     0.62
## peer_groupD1a                     0.17      0.25    -0.33     0.66
## peer_groupD1b                    -0.30      0.44    -1.17     0.56
## IsrvproptotMmeansrvproptotD10     0.11      0.03     0.05     0.16
## IfemalepropM0.5MU10              -0.14      0.07    -0.27    -0.00
## Iage65M0.5MU10                   -0.03      0.04    -0.11     0.04
## ruralR                           -0.31      0.14    -0.58    -0.01
##                               Eff.Sample Rhat
## Intercept                            174 1.02
## cYear                               1000 1.00
## peer_groupB                          231 1.03
## peer_groupC1                         157 1.04
## peer_groupC2                         187 1.03
## peer_groupD1a                        187 1.02
## peer_groupD1b                        513 1.02
## IsrvproptotMmeansrvproptotD10        847 1.00
## IfemalepropM0.5MU10                  800 1.01
## Iage65M0.5MU10                       627 1.00
## ruralR                               244 1.02
##
## Samples were drawn using sampling(NUTS). For each parameter, Eff.Sample
## is a crude measure of effective sample size, and Rhat is the potential
## scale reduction factor on split chains (at convergence, Rhat = 1).
```

```
mdls <- mget(ls(pattern = "^m_"))
saveRDS(mdls, here("hosp-variation", "output", "hosp_Var_models.rds"))
```
